# Quantifying the impact of the Grain-for-Green Program on ecosystem service scarcity value in Qinghai, China

**DOI:** 10.1038/s41598-023-29937-7

**Published:** 2023-02-20

**Authors:** Yu Hu, Shidong Zhang, Yu Shi, Luo Guo

**Affiliations:** 1 College of Life and Environmental Sciences, Minzu University of China, Beijing, 100081 China; 2 Research Center for Eco-Environmental Sciences, Chinese Academy of Sciences, The State Key Laboratory of Urban and Regional Ecology of China, Beijing, 100085 China

**Keywords:** Ecosystem services, Environmental economics

## Abstract

Studying the impact of large-scale ecological projects, such as the Grain-for-Green Program (GGP), on ecosystem services (ES) is currently a frontier and hot topic of ecological research. The GGP can directly change land use and land cover, thus affecting ES. By comparing the changes of ecosystem service value (ESV) and ecosystem service scarcity value (ESSV) in Qinghai before and after the implementation of the GGP, this paper clarified the impact of the GGP on Qinghai from the angles of ecology and economics. This paper quantified and evaluated the land use dynamics, ESV, and ESSV in Qinghai from 1995 to 2020. The results showed that in the past 25 years, the total annual Normalized Difference Vegetation Index (NDVI) of Qinghai showed a trend of sustained growth. From 1995 to 2020, the ESV increased by 6.80%. After considering supply and demand, the ESSV showed a continuous upward trend, increasing by 719.38%. After implementation of the GGP, the increase of NDVI inhibited the increase of the ESSV. These findings from evaluation of the effect of the GGP implementation provide a theoretical basis for future policy implementation and, in particular, a reference for the evaluation of the ESV and the ESSV in Qinghai.

## Introduction

The Grain-for-Green Program (GGP) is an ecological construction project with the most robust policy, the largest investment, the broadest coverage, and the greatest level of public engagement in China, if not the globe^1^. The purpose of the GGP is to improve the ecological environment and to transform low-quality farmland into forest and grassland, the GGP plays a role in restoring vegetation^2^, so as to establish a community of human and natural life^3^. In 1999, some provinces in China took the lead in carrying out a pilot GGP, and since then it has been fully launched nationwide^4^. By 2020, China had invested 535.3 billion yuan and implemented the GGP in 34.8 million hm^2^, representing 40% of all afforestation regions from national key projects within the same period. In addition, the GGP has improved the harsh ecological environment in Western China. Proper political decision-making is helpful for ecological projects to exert their maximum benefits, so more and more attention is paid to the research related to ecological restoration projects around the world, the research focuses on the evaluation related to the implementation effect of the policy. Policy evaluation methods can be divided from multiple perspectives, including: Contrastive analysis, Cost–Benefit Analysis, Causal inference method, etc. The comparative analysis method is to compare the research object with the benchmark or reference frame to evaluate the effectiveness of the policy^5^. The cost–benefit analysis method is a policy evaluation method through comparative analysis of the costs and benefits generated in the process of policy operation^6^. The causal interference method can describe and explain social phenomena, and establishing a credible causal relationship between a public policy and its impact is of great significance for policy evaluation. If the intervention in the experiment is randomly assigned, it is a experimental design, otherwise it is a quasi-experimental design^7^. The main application categories of experimental design in policy evaluation include laboratory experiment, investigation experiment and field experiment^8^. And quasi-experimental design include the difference-in-differences (DID) method^9^, synthetic control method (SCM)^10^ and regression discontinuity design^11^, etc. In this paper, the contrastive analysis is selected to quantitatively analyze the changes of ESSV before and after GGP implementation.

The research angle of ecological projects have shown a diversified trend, Bronwen et al.’ s research on restoration projects on the California Central Coast showed that the restoration activity is consistent with ecological need, concentrated in human settlements and near restoration organizations^12^; Milchakova et al.’s research showed that the implementation of the ecological framework project will help to reduce the risk of losing the biological and landscape diversity of Sevastopol City^13^; Roy et al. expounded design, governance, and outcomes of the Flagstaff Watershed Protection Project^14^. In China, the research related to ecological projects mainly focuses on GGP. According to whether the manifestation of the research object is direct or not, the research related to GGP effect evaluation can be divided into direct research and indirect research. Direct research reveals the influence of GGP on vegetation coverage^15^, climate change^16^, land use^17^ and various social phenomena^18,19^. These factors have the characteristics of direct expression and easy observation. Indirect research reveals the influence of GGP on ecological health^20^, ecological fragility^21^ and ES on the basis of direct research, among which the research on ES occupies the mainstream position.

Qinghai launched the GGP in 2000^22^. Qinghai is an underdeveloped and once a deep poverty-stricken area and also the source of the three rivers and the Chinese water tower^23,24^. Ecosystems of Qinghai produce a large number of products and provide huge number of ecosystem services (ES)^25^. Therefore, Qinghai shoulders not only the task of ecological protection, but also the task of economic development^26^. Its ecological environment condition is closely related to the sustainable development of social economy^27^, human being's living and producing. The stability of Qinghai’s ecological environment is of great importance for China and even Southeast Asia^24^. However, with social progress, the ecological environment in Qinghai has also been damaged to varying degrees^28^. GGP construction areas are all over Qinghai, including 41 counties (cities, districts), accounting for 64.4% of the province's total area. GGP has the greatest influence on Qinghai and is the main ecological policy of Qinghai and even China^29^. The implementation of the GGP can significantly change the land use dynamics, and land use and land cover (LULC) determines the maintenance of ES^30^. Hence, besides ecological and natural benefits^31,32^, research by Cao et al. showed that the GGP increased net benefits for citizens of China, and this suggests that such large-scale ecological projects are potentially profitable^33^, the implementation of the GGP can also create monetary and social benefits^34,35^. Thus, it is necessary to explore the impact of the GGP on ES in Qinghai.

ES refer to various irreplaceable environmental conditions and products for the existence and development of mankind^36,37^, ecosystems and related environmental processes are the foundation of human existence, from which mankind can benefit directly or indirectly^38,39^. Land use is the main link between ES and human activities. Under the indirect influence of LULC, ecological engineering is often accompanied by the change of land use pattern^40^. With the implementation of GGP, the land use pattern has changed significantly, which has greatly changed the supply of various ES, and ES has changed to a great extent^41^. And changes in LULC will directly change the ecosystem type and its area and spatial distribution^42^. Under the background of human-land interaction transformation, land use change scenario analysis has become an important means to quantify the response of ES to land use policies and quantitative analysis of ES is an critical step to realize the concept of land use administration and decision-making^43^. In addition, quantitative analysis of the ecosystem services value (ESV) can be performed to evaluate the influence of LULC changes on ES^44^. Consuming goods or services can bring well-being to mankind, and the economic value, which can be evaluated using market and non-market valuation techniques, can be used as a measure such benefits^45^. Moreover, ESV assessment can provide a basis for evaluating the effectiveness of ecological projects^46^. Following the first evaluation of global ESV by Costanza et al.^38^, there has been an upsurge of evaluating ESV, and a variety of evaluation methods have been formed so far, including the direct and indirect evaluation the ESV based on market theory. In addition, quantitative modeling also plays a vital role, including: biophysical, empirical, GIS-based models and so on^47^. The current research mainly uses the indirect evaluation method based on market theory^48^, which is based on value equivalent method.

ES are products that can be consumed by mankind^36,37^, ecosystems can provide a variety of services for human beings^49^. As the society develops, increasing growth of the human social economy promotes the consumption of the supply of ES. Therefore, the demand for ES will also continue to grow. However, the ability of the ESV to comprehensively reflect the changes in a research area is limited; specifically, the ESV cannot reflect change of supply and demand caused by social economy, Shi et al. evaluated the supply and demand situation and to identify the supply and demand areas of ES in Shanghai^50^; the research of Zoderer et al.^51^ expounds the views of stakeholders on supply and demand of ES bundle; and Bryan et al.^52^ used scarcity value to make up for the defects of traditional ESV evaluation. The ecosystem service scarcity value (ESSV) can reflect the fluctuation of ESV caused by the change of supply and demand. The scarcity coefficient of supply and demand can be introduced into ESV evaluation to quantify the impact of GGP implementation on the ESV from the angle of economics. Ecological restoration initiatives around the world have greatly improved the provision of ES^33^. Only when the supply level of ES meets human needs, will ES reach a balance. Therefore, increasing the supply of ES can alleviate the growth of the ESSV. Yet the strongest effects on ecosystem service scarcity value occur in landscapes that are subject to significant supply-side and demand-side dynamics such as areas disturbed by human beings^52^. The GGP is the positive protection of forest and grassland by human beings by interfering with the natural environment, the promotion of the GGP has gradually improved the regional ecological environment, expanded the area of forest and grassland, and correspondingly increased the supply of ES^33^. Qinghai is one of the key areas of implementing the GGP in China^39^. Therefore, taking Qinghai as the research area to explore the impact of supply and demand dynamics on ecosystem service scarcity value has important practical significance.

At present, the assessment of the influence of the GGP on ES is mostly rooted in the analysis of physical supply, rather than the effect of changes in supply and demand on the ESV from an economic point of view. The total ESV may change when the scarcity value is taken into account. To sum up, including ESSV when measuring the total ESV can allow simultaneous evaluation of the effect of LULC changes on ES from ecological and economic perspectives^2^. Further exploring the effect of LULC changes on the ESV and ESSV before and after GGP implementation can provide reference for relevant land use policies in Qinghai.

This study quantified the impact of the GGP on the ESSV in 1995–2020 by comparing the changes of ESSV before and after the implementation of the GGP. The conclusions drawn in this paper can provide effective data support and reasonable policy guidance for the comprehensive management and subsequent implementation of the GGP in areas not limited to Qinghai.

## Methods

### Study area

**Figure 1:**
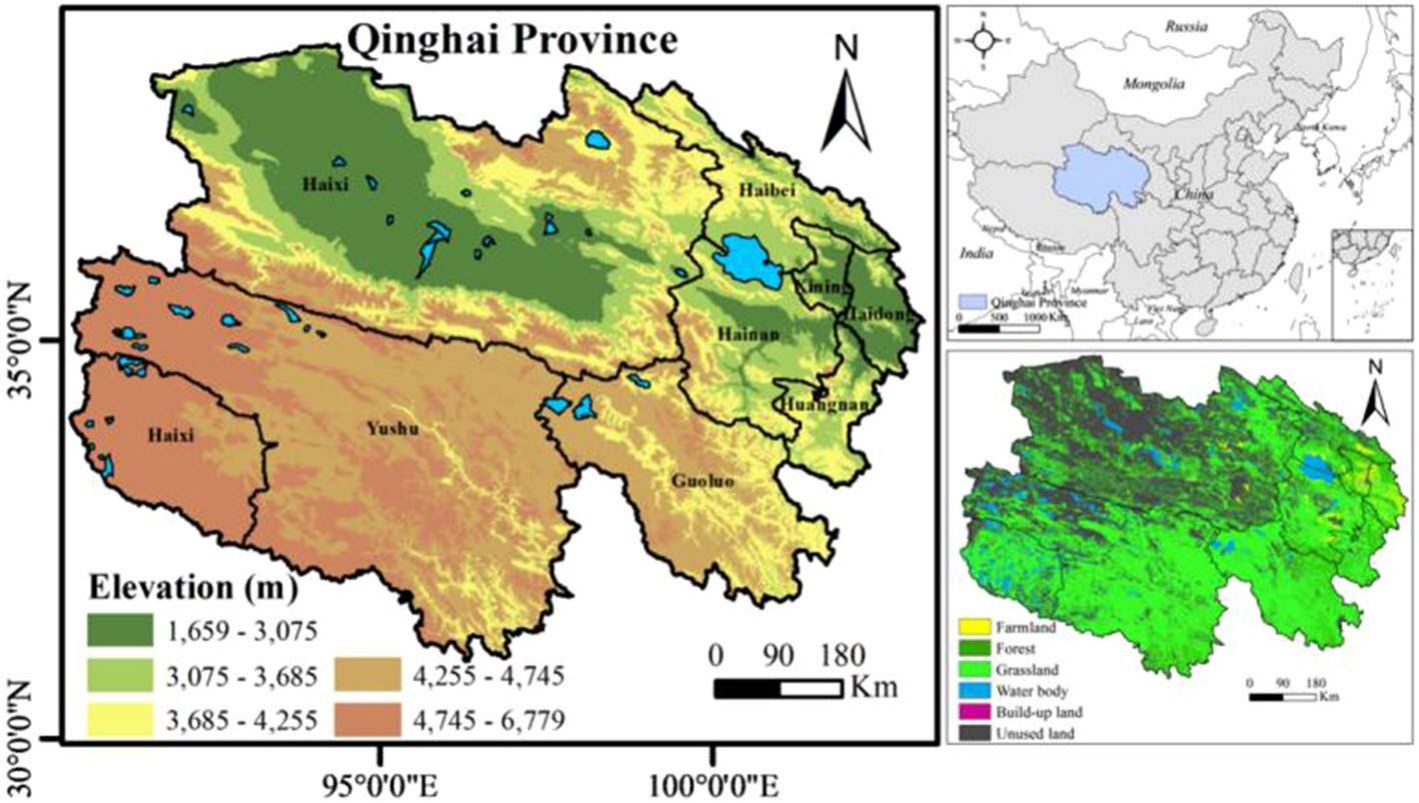
Location of the study area (made in ArcGIS 10.4.1, https://www.esri.com/zh-cn/arcgis/products/arcgis-desktop/resources).

Qinghai Province (31°39′–39°19′ N, 89°35′–103°04′ E) is located in northwest inland China and covers an area of 7.2 × 10^5^ km^2^^53,54^. Qinghai has complex and diverse landforms^55^, the eastern part is mountainous, while the western part is plateau and basin^56^ (Fig. 1). Qinghai is located in the Qinghai-Tibet Plateau^57^, the largest plateau in China and the highest altitude in the world, with an average altitude of more than 4000 m, which belongs to plateau continental climate^58^. The Qinghai-Tibet Plateau is the birthplace of many big rivers in East Asia, Southeast Asia and South Asia. There are many lakes on the plateau, including Namtso and Qinghai Lake. In recent years, the economy of Qinghai-Tibet Plateau has continued to grow, and the population scale has been expanding^40^. During the study period, the population of Qinghai increased from 4.48 million to 5.93 million. The economic development situation continued to improve. In 2020, Qinghai's annual GDP was 43.56 US$ billion, an increase of 1.5% over that of the previous year at comparable prices. The values of primary, secondary, and tertiary industries all showed an increasing trend.

### Data collection

The land use data from the Resource and Environment Science Data Center (RESDC) of the Chinese Academy of Sciences, with a spatial resolution of 1000 m × 1000 m. Based on China's national standard land use classification, LULC types include farmland, forest, grassland, water body, build-up land and unused land. The annual Normalized Difference Vegetation Index (NDVI) spatial distribution data set is derived from http://www.vito-eodata.be and the spatial resolution is 1 km. The annual NDVI data is the maximum value of monthly NDVI in 1–12 months of each year. Social and economic data are from National Bureau of Statistics of China^59^ and Statistics Bureau of Qinghai^60^.

### Methods

#### Geo-information Tupu change analysis based on GIS

Geo-information Tupu combines remote sensing images and GIS, and makes full digital detailed analysis by using the thinking idea of Geo-information Tupu, so as to describe the evolution characteristics of the research object. For example, describe LULC change and reveal its internal law of change^61,62^. The details of geographical phenomena and the changes of geographical processes should be revealed in the form of graph-spectrum coupling to the deep geographical synthesis and geographical pattern^63^.

The Tupu model^64^ of land use change in Qinghai is established by map algebraic superposition of data in six periods through ArcGIS^65^. The formula is:

1 $$ T \, = \, \gamma_{1} \times \, 10^{n - 1} + \, \gamma_{2} \times \, 10^{n - 2} + \, \cdot \, \cdot \, \cdot \, + \, \gamma_{n} \times \, 10^{n - n} $$

where T is the Tupu unit code value of Tupu mode characteristics during the study period; Y_n_ is the unit code value of the land use map in a certain year; n is the number of land use type.

Tupu code of AB means that the land use type had converted from A to B. Spatial segregation index of land use change can clearly express land use pattern change^66^. The formula of change rate and spatial separation degree is:

2 $$ R_{ij} = A_{ij} \times 100\% /\sum\limits_{i = 1}^{n} {\sum\limits_{j = 1}^{n} {A_{ij} } } $$

3 $$ {{S_{ij} = \frac{1}{2} \times \sqrt {D_{ij} /\sum\limits_{i = 1}^{n} {\sum\limits_{j = 1}^{n} {A_{ij} } } } } \mathord{\left/ {\vphantom {{S_{ij} = \frac{1}{2} \times \sqrt {D_{ij} /\sum\limits_{i = 1}^{n} {\sum\limits_{j = 1}^{n} {A_{ij} } } } } {A_{ij} /\sum\limits_{i = 1}^{n} {\sum\limits_{j = 1}^{n} {A_{ij} } } }}} \right. \kern-0pt} {A_{ij} /\sum\limits_{i = 1}^{n} {\sum\limits_{j = 1}^{n} {A_{ij} } } }} $$

where, *R*_*ij*_ is change ratio, which represents the ratio of the Tupu unit area of individual to all transferred land use change Tupu in all regions. *S*_*ij*_ is space separating degree, which reflects the dispersion degree of land use Tupu unit. *D*_*ij*_, *A*_*ij*_ present the number of Tupu unit and area of land use type i of t changes to land use type j of (t + ∆t), and n is the number of land use type.

#### Landscape pattern index

Patch Density (PD), Landscape Shape Index (LSI), Largest Patch Index (LPI) and Shannon’s Diversity Index (SHDI) were selected. These indexes can effectively reflect the landscape structure in Qinghai^66^. The landscape pattern indices were analyzed by Fragstats 4.2 software.

#### Calculation of the ESV

The ESV was evaluated by the method proposed by Xie et al.^67^. The 1/7 of the economic value of food production service from farmland is defined as the per-unit ESV^68^. On the basis of the average grain price and average annual grain yield in Qinghai from 1995 to 2020, the unit ESV in Qinghai was calculated, the formula is:

4 $${ESV}_{s,k,t}={\alpha }_{k,t}\times {VC}_{s,k}$$

where ${\alpha }_{k,t}$ refers to the area of land use type k at time t. ${VC}_{s,k}$ indicates the per-unit value coefficient of ES (Table 1), and k is land use type; t ∈ {1995, 2000, 2005, 2010, 2015, 2020}.

**Table 1:** The Qinghai’s per-unit value coefficient of ES.

Ecosystem service types	Farmland	Forest	Grassland	Water body	Build-up land	Unused land
Air quality	1.39 × 10^–7^	9.71 × 10^–7^	2.22 × 10^–7^	0.00	− 6.72 × 10^–7^	0.00
Climate regulation	2.47 × 10^–7^	7.49 × 10^–7^	2.50 × 10^–7^	1.28 × 10^–7^	0.00	0.00
Water supply	1.67 × 10^–7^	8.88 × 10^–7^	2.22 × 10^–7^	5.66 × 10^–6^	− 2.08 × 10^–6^	8.33 × 10^–9^
Soil retention	4.05 × 10^–7^	1.08 × 10^–6^	5.41 × 10^–7^	2.78 × 10^–9^	5.55 × 10^–9^	5.55 × 10^–9^
Waste treatment	4.55 × 10^–7^	3.64 × 10^–7^	3.64 × 10^–7^	5.05 × 10^–6^	− 6.83 × 10^–7^	2.78 × 10^–9^
Biodiversity services	1.97 × 10^–7^	9.05 × 10^–7^	3.03 × 10^–7^	6.91 × 10^–7^	9.44 × 10^–8^	9.44 × 10^–8^
Food production	2.78 × 10^–7^	2.78 × 10^–8^	8.33 × 10^–8^	2.78 × 10^–8^	2.78 × 10^–9^	2.78 × 10^–9^
Raw materials	2.78 × 10^–8^	7.22 × 10^–7^	1.39 × 10^–8^	2.78 × 10^–9^	0.00	0.00
Recreation and culture	2.78 × 10^–9^	3.55 × 10^–7^	1.11 × 10^–8^	1.20 × 10^–6^	2.78 × 10^–9^	2.78 × 10^–9^
Total	1.92 × 10^–6^	6.06 × 10^–6^	2.01 × 10^–6^	1.28 × 10^–5^	− 3.33 × 10^–6^	1.17 × 10^–7^

Unit: 2020US$ Billion/hm^2^.

#### Calculation of the ESSV

Based on the mould of Bryan et al.^52^, ESSVs assessment were divided into: Naive (scenario 1), supply elasticity (scenario 2), demand elasticity (scenario 3) and demand and supply elasticity (scenario 4)^69^. Division of four scenarios can clearly reflect the effects of supply and demand dynamics on the ESSV^70^. Naive of ES means that only the material supply value is considered. The comparison of the ESSV under the four scenarios is based on scenario 1. In view of the influence of supply and demand elasticity on ES^70^ (Table 2), it is necessary to quantify the impact of private products or private products on supply and demand respectively. Scenarios 2, 3 and 4 respectively reflect the impact of supply, demand and the combination of supply and demand on the ESSV by increasing supply price elasticity, demand price elasticity and simultaneously increasing supply and demand price elasticity. The data in 1990 is calculated as 1 in proportion, and all calculations are based on this. The formula used is as follows:

**Table 2:** Relative changes of scarcity value under four scenarios.

Scenarios	Demand (${\Delta P}_{s,t1}^{Dem}$)		Supply (${\Delta P}_{s,t1}^{Sup}$)	
	Private good	Public good	Private good	Public good
Naive	0.00	0.00	0.00	0.00
Demand	0.25	0.80	0.00	0.00
Supply	0.00	0.00	0.25	0.50
Demand + Supply	0.25	0.80	0.25	0.50

5 $${ESSV}_{s,k,t1}={\alpha }_{k,t1}\times {VC}_{s,k}\times (1+{\Delta p}_{s,t1}^{Sup}+{\Delta p}_{s,t1}^{Dem})$$

where ${ESSV}_{s,k,t1}$ is the ESSV at time t1, t1 ∈ {1995, 2000, 2005, 2010, 2015, 2020} (Same as below). ${\Delta p}_{s,t1}^{Sup}$, ${\Delta p}_{s,t1}^{Dem}$ respectively represent supply-driven and demand-driven change in scarcity value of each ES at time t1. Therefore:

6 $${\Delta p}_{s,t1}^{Sup}={\Delta Q}_{s,t1}^{Sup}\times {\Delta P}_{s,t1}^{Sup}$$

7 $${\Delta Q}_{s,t1}^{Sup}=-\frac{{\sum }_{k\in K}{ESV}_{s,k,t1}^{Naive}-{\sum }_{k\in K}{ESV}_{s,k,t0}^{Naive}}{{\sum }_{k\in K}{ESV}_{s,k,t0}^{Naive}}$$

where, ${\Delta Q}_{s,t1}^{Sup}$ represents the fixed value of the proportionate change in the supply for ES between t0 and t1, t0 = 1990 (Same as below). ${\Delta p}_{s,t1}^{Sup}$ represents the relative change in the value of scarcity after a 100% reduction in supply (Table 3). K represents six land use types.

**Table 3:** Changes in the proportion of willingness to pay in Qinghai.

ES	The proportion change in the willingness to pay (${\Delta Q}_{s,t}^{Dem}$)					
	1990	1995	2000	2005	2010	2015
Food production	0.00	0.11	0.15	0.48	1.19	1.83
Raw material	0.00	0.05	0.10	0.25	0.55	0.94
Air quality	0.00	0.79	1.38	3.50	7.52	12.44
Climate regulation	0.00	0.79	1.38	3.50	7.52	12.44
Water supply	0.00	0.52	0.90	2.35	5.25	9.11
Waste treatment	0.00	0.02	0.03	0.09	0.21	0.38
Soil retention	0.00	0.52	0.90	2.35	5.25	9.11
Biodiversity services	0.00	0.52	0.90	2.35	5.25	9.11
Recreation and culture	0.00	1.60	2.79	7.27	16.32	28.51

8 $${\Delta p}_{s,t1}^{Dem}={\Delta Q}_{s,t1}^{Dem}\times {\Delta P}_{s,t1}^{Dem}$$

9 $${\Delta Q}_{s,t1}^{Dem}=\frac{({WTP}_{s,t1}-{WTP}_{s,t0})}{{WTP}_{s,t0}}$$

10 $${WTP}_{s,t}={POP}_{t}\times {GDP}_{t}\times {\varepsilon }_{s,t}$$

$${\Delta Q}_{s,t1}^{Dem}$$ represents the fixed value of the proportionate change in the demand for ES between t0 and t1. $${\Delta P}_{s,t1}^{Dem}$$ represents the relative change in the value of scarcity after a 100% reduction in demand (Table 3). $${WTP}_{s,t}$$ represents the willingness of people to pay for ES at time t in Qinghai. $${\mathrm{POP}}_{\mathrm{t}}$$ represents the total population at time t in Qinghai. $${GDP}_{t}$$ represents the real Gross Domestic Product per capital, which adjusted for inflation to 2020 dollars (US$ /cap) (Table 4). $${\varepsilon }_{s,t}$$ refers to the income elasticity for each ES at time t (Table 5).

**Table 4:** Population and real per-capita GDP in Qinghai.

Year	GDP (US$ Billion)	CPI* (adjustment factor)	Real GDP (2020 US$ Billion)	Population (Million)	Real GDP per capita (2020 US$ Billion)
1990	1.01	101.98	0.99	4.48	220.98
1995	2.43	116.59	2.08	4.81	432.43
2000	3.82	97.01	3.94	5.17	762.09
2005	7.24	97.07	7.46	5.43	1373.85
2010	16.58	102.40	16.19	5.63	2875.67
2015	29.15	100.17	29.10	5.77	5043.33
2020	43.62	100	43.62	5.93	7355.82

**Table 5:** Income elasticity of demand.

ES	The expression of income elasticity	Income elasticity (${\varepsilon }_{s,t}$)					
		1990	1995	2000	2005	2010	2015
Food production	Engel Coefficient	0.58	0.51	0.44	0.42	0.42	0.37
Raw material	*y* = $$- $$*3E−06x* + *0.3276*	0.33	0.33	0.33	0.32	0.32	0.32
Air quality	*y* = *2E−10x*^*2*^*−4E−05x*+*1.2649*	1.23	1.23	1.24	1.21	1.17	1.12
Climate regulation	*y* = *2E−10x*^*2*^*−4E−05x*+*1.2649*	1.23	1.23	1.24	1.21	1.17	1.12
Water supply	*y* = *0.45*	0.45	0.45	0.45	0.45	0.45	0.45
Waste treatment	*y* = *1E−05x*+*0.1814*	0.19	0.19	0.19	0.19	0.20	0.22
Soil retention	*y* = *0.3*	0.30	0.30	0.30	0.30	0.30	0.30
Biodiversity services	*y* = *0.38*	0.38	0.38	0.38	0.38	0.38	0.38
Recreation and culture	*y* = *9E−06x*+*1.7477*	1.75	1.76	1.75	1.76	1.77	1.78

#### Cold hotspots analysis

The Getis-Ord Gi* index was used to analyze the spatial aggregation degree of the ESSV change. The formula used is as follows:

11 $$ Gi^{*} = \frac{{\sum\limits_{j = 1}^{n} {w_{ij} } x_{j} - \overline{X}\sum\limits_{j = 1}^{n} {w_{ij} } }}{{s\sqrt {\left[ {n\sum\limits_{j = 1}^{n} {w_{ij}^{2} } - \left( {\sum\limits_{j = 1}^{n} {w_{ij} } } \right)^{2} } \right]/(n - 1)} }} $$

12 $$ \overline{X} = \frac{1}{n}\sum\limits_{j = 1}^{n} {x_{j} } $$

13 $$ S = \sqrt {\left( {\frac{1}{n}\sum\limits_{j = 1}^{n} {x_{j}^{2} } - \overline{X}^{2} } \right)} $$

where, $Gi^{*}$ is the output statistical Z-score; $x_{j}$ is the ESSV change of space unit j; $w_{ij}$ is the spatial weight between adjacent space units i and j.

## Results

### Changes in LULC

**Figure 2:**
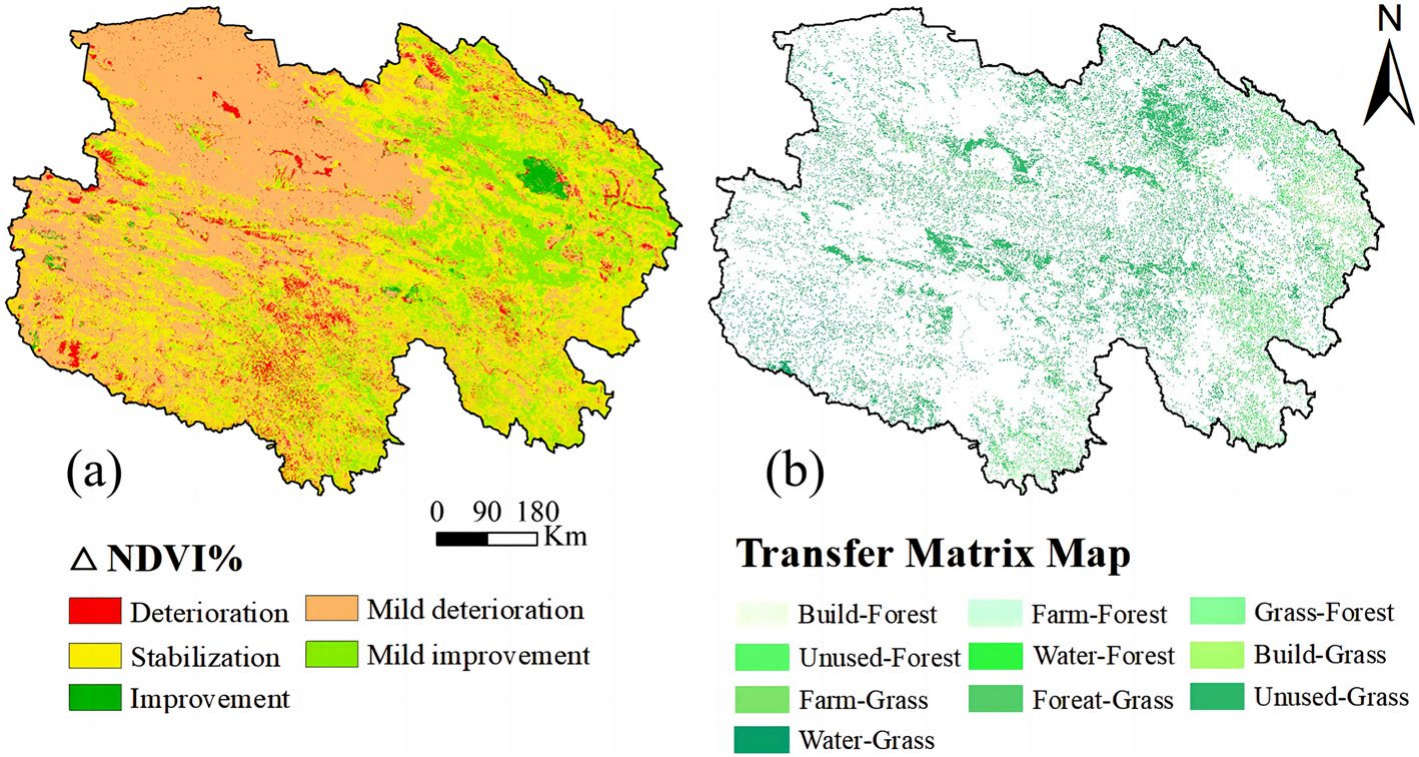
(**a**) Changes in the Normalized Difference Vegetation Index (NDVI) from 1995 to 2020; (**b**) Types of land-use transfer from 1995 to 2020 (made in ArcGIS 10.4.1, https://www.esri.com/zh-cn/arcgis/products/arcgis-desktop/resources).

A change in NDVI reflects the dynamic changes of forest and grassland productivity, and visualization of NDVI reveals the overall change of LULC (Fig. 2a). NDVI continuously increased from 1995 to 2020. The total NDVI values in 1995, 2000, 2005, 2010, 2015, and 2020 were 0.23, 0.24, 0.25, 0.25, 0.27, and 0.27 respectively, indicating that the vegetation coverage showed an upward trend in Qinghai. Analysis of the spatial distribution of NDVI revealed that the green space coverage in Qinghai gradually decreased from southeast to northwest.

The change in LULC in Qinghai is shown in Fig. 2b. The transfer of LULC from other types to forest and grassland was the most common LULC transfer type from 1995 to 2020. The area of farmland transferred to forest and grassland was 3016 km^2^, and the area of other LULC types transferred to forest and grassland was 83,204 km^2^. According to the analysis of spatial distribution, forest and grassland were mainly distributed in eastern and central Qinghai.

### Changes in temporal and spatial heterogeneity in landscape patterns

PD, LSI, LPI, and SHDI can reveal the changes in temporal and spatial heterogeneity of the landscape pattern index from 1995 to 2020. The temporal changes in PD, LSI, and SHDI values were similar. Before the GGP was implemented, PD, LSI, and SHDI values increased significantly. After GGP implementation, PD, LSI, and SHDI values changed little, then decreased until 2015. LPI values and the other three landscape index values showed opposite trends. Before GGP implementation, LPI values decreased significantly. After GGP implementation, LPI values did not changed, then increased until 2015.

**Figure 3:**
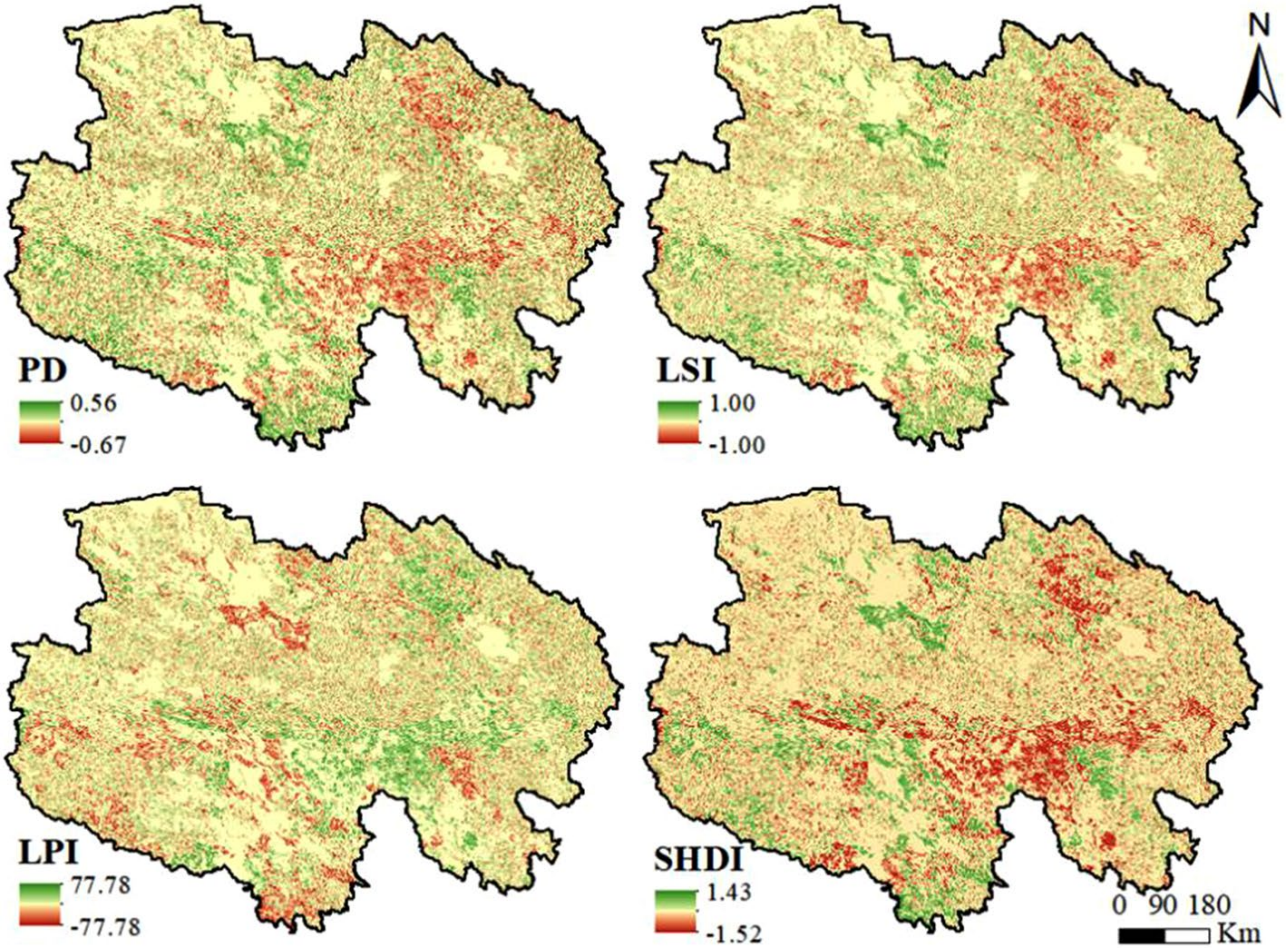
Changes in landscape pattern index values from 1995 to 2020 (made in ArcGIS 10.4.1, https://www.esri.com/zh-cn/arcgis/products/arcgis-desktop/resources).

Spatial heterogeneity analysis of landscape patterns showed that the changes in PD, LSI and SHDI values were similar during the study period (Fig. 3). The areas where these values increased are mainly concentrated in the Buha River Basin of Haixi, which is situated in the northeast of Qinghai; the Yellow River Basin of Hainan, located in the east of Qinghai; the Yellow River Basin and around Zhaling Lake, Erling Lake, and Donggi Cuona Lake of Guoluo, which is situated in the southeast of Qinghai; and the headwaters of the Yangtze River and the Yellow River of Yushu, which is situated in the southwest of Qinghai. The areas where PD, LSI, and SHDI values decreased are mainly concentrated around the West Dabsan Lake, East Dabsan Lake, North Hobsun Lake, and South Hobsun Lake of Haixi, which are situated in the west of Qinghai; the Yellow River Basin in the center of Guoluo; and some areas in the southwest of Qinghai. The change of LPI values is opposite to the changes of the spatial distributions of PD, LSI, and SHDI values.

### Temporal and spatial heterogeneity changes in the ESV

The temporal changes of ESV is shown in Table 6. From 1995 to 2020, the ESV of Qinghai first decreased and then increased. Before GGP implementation, the ESV decreased by 0.89%. After GGP implementation, the ESV kept rising, with an overall increase of 7.76%.

**Table 6:** The ecosystem service values (ESVs) from 1995 to 2020 (unit: 2020 US$ Billion).

Ecosystem service types	1995	2000	2005	2010	2015	2020
Air quality	76.88	76.97	76.75	76.67	76.39	79.50
Climate regulation	83.16	83.22	83.13	83.14	83.11	86.95
Water supply	179.10	183.22	184.86	185.75	187.83	206.31
Soil retention	165.11	164.61	164.33	164.30	164.10	171.64
Waste treatment	196.24	199.36	200.93	201.84	203.96	222.91
Biodiversity services	144.03	127.35	127.46	127.56	127.77	132.23
Food production	24.80	24.66	24.63	24.63	24.61	25.88
Raw materials	17.61	18.15	18.13	18.13	18.12	182.17
Recreation and culture	32.11	33.28	33.72	33.95	34.53	37.85
Total value	919.04	910.82	913.93	915.97	920.41	981.49

The spatial changes of ESV was analyzed by the municipal administrative division of Qinghai. As far as the administrative division is concerned, the total ESV of Haixi, which is situated in the west of Qinghai, was the highest and that of Xining, which is situated in the east of Qinghai, was the lowest. The total ESV of various cities and autonomous prefectures was positively correlated with the area. According to the average ESV of various cities and autonomous prefectures, Haibei, which is situated in the northeast of Qinghai, had the highest ESV and Haixi had the lowest. The change of ESV in Guoluo was consistent with that in Qinghai. Before GGP implementation, it decreased, but after GGP implementation, it increased. The ESV in Haixi showed continuous growth, with an increase of 27.90% from 1995 to 2020.

### Changes in the ESSV

#### Temporal change of the ESSV

**Figure 4:**
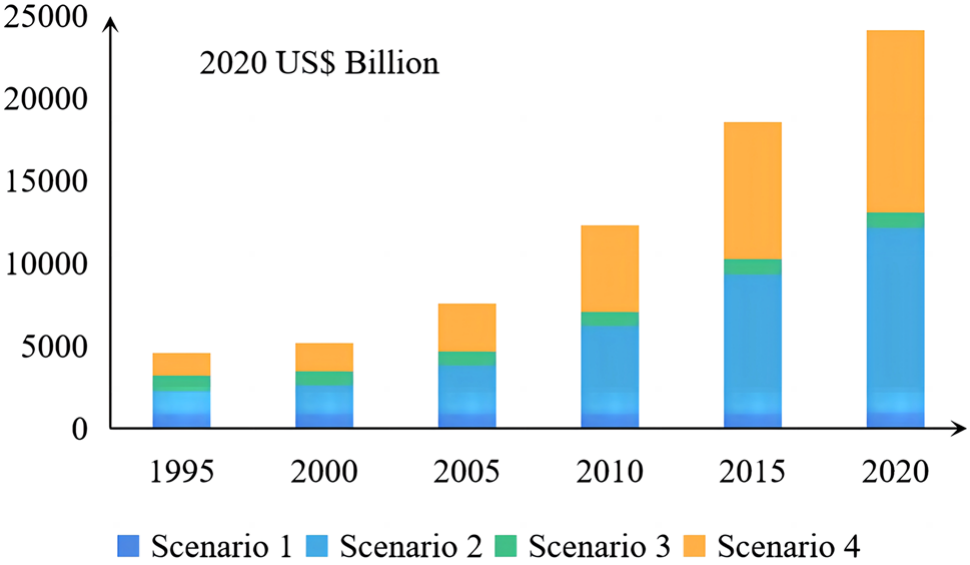
The ecosystem service scarcity values (ESSVs) under different scenarios (unit: 2020 US$ Billion).

**Figure 5:**
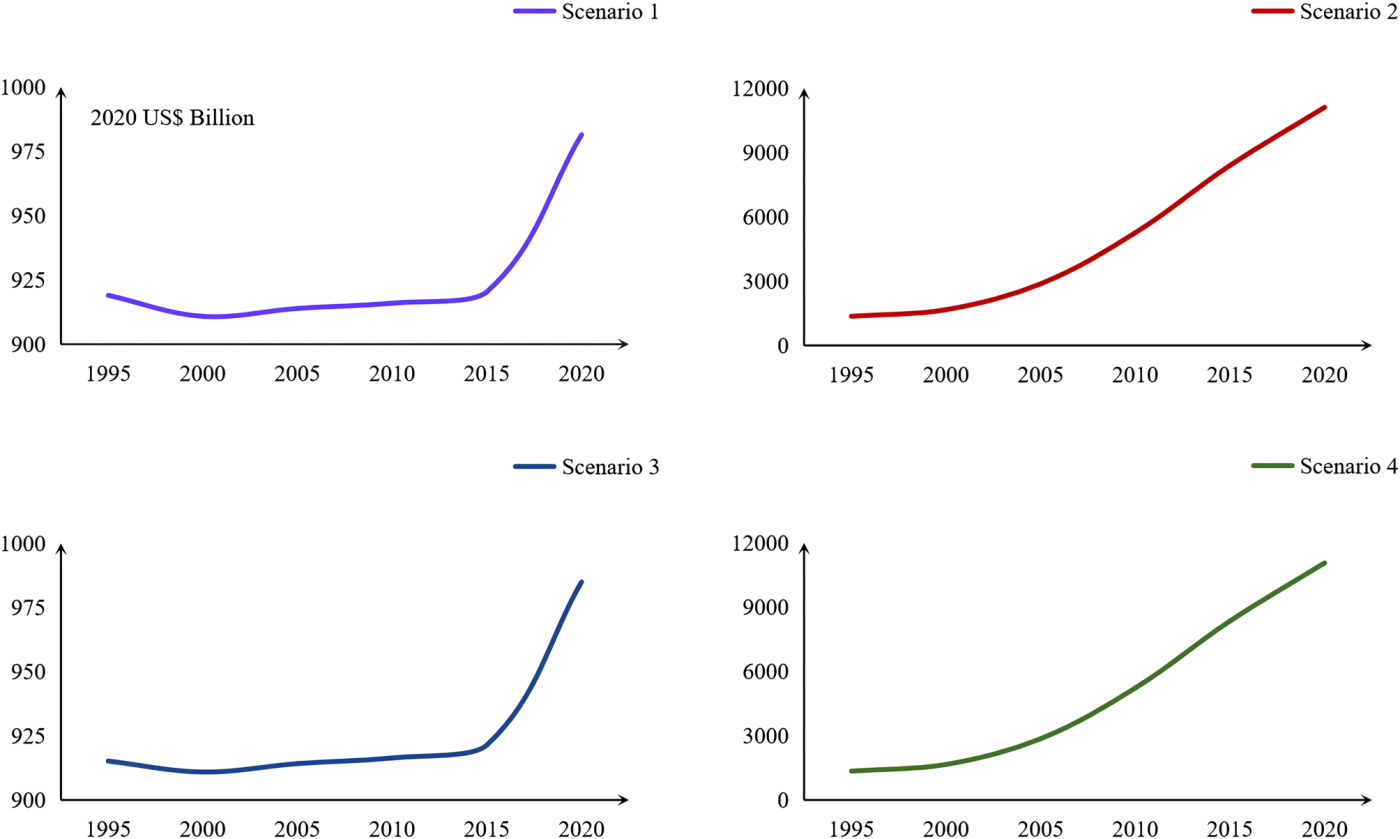
The respective curves of ESSVs under scenario 1–4 (unit: 2020 US$ Billion).

The ESSVs for the four scenarios from 1995 to 2020 are shown in Fig. 4. The average ESSVs for the scenario ranked as follows: 2 > 4 > 3 > 1. The ESSVs for scenarios 2, 3, and 4 were 451.45%, 448.96%, and 0.03% higher than scenario 1, respectively. Figure 5 intuitively reflected the respective change trends of ESSVs under scenarios 1–4. It can be seen that the change trend of scenario 1 and 3 is similar, while that of scenario 2 and 4 is similar. Significant increase in ESSVs when considering demand, indicating that human demand for ES strongly affected the ESSV. To determine the influence of supply on the ESSV visually, we compared scenario 2 with scenario 4. Before GGP implementation, the growth rate of scenario 4 was higher than that of scenario 2, but from 2000 to 2010 it was lower, indicating that the GGP increased the role of supply and delayed the growth of the ESSV. However, the growth rate of scenario 4 was no longer lower than that of scenario 2 after 2010; the increase of the ESSV caused by increased demand is greater than the decrease of the ESSV caused by increased supply.

**Figure 6:**
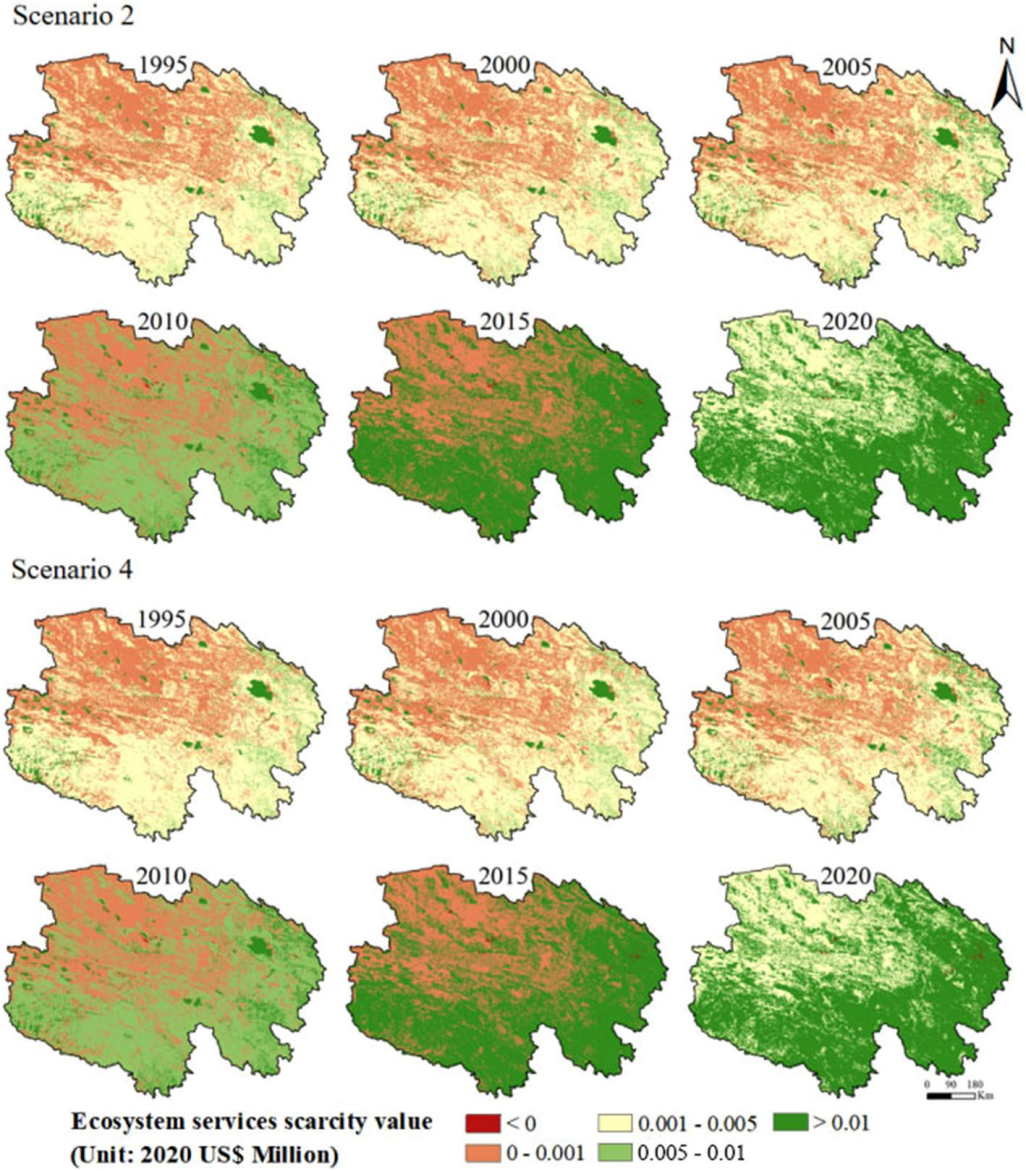
Spatial distribution of the ESSVs for scenario 2 and scenario 4 (made in ArcGIS 10.4.1, https://www.esri.com/zh-cn/arcgis/products/arcgis-desktop/resources).

Changes in the spatial distribution of ESSVs during the study period are shown in Fig. 6. Under scenarios 2 and 4, the spatial distributions of the ESSV (both characterized by a gradual decrease from the southeast to northwest) were not significantly different, indicating that there was no obvious inhibition of the growth of the ESSV by supply factors. Before 2015, the ESSV in Qinghai Lake was higher than that in neighboring areas, and the gap with the surrounding areas gradually narrowed after 2015.

#### Change in the spatial heterogeneity of the ESSV

**Figure 7:**
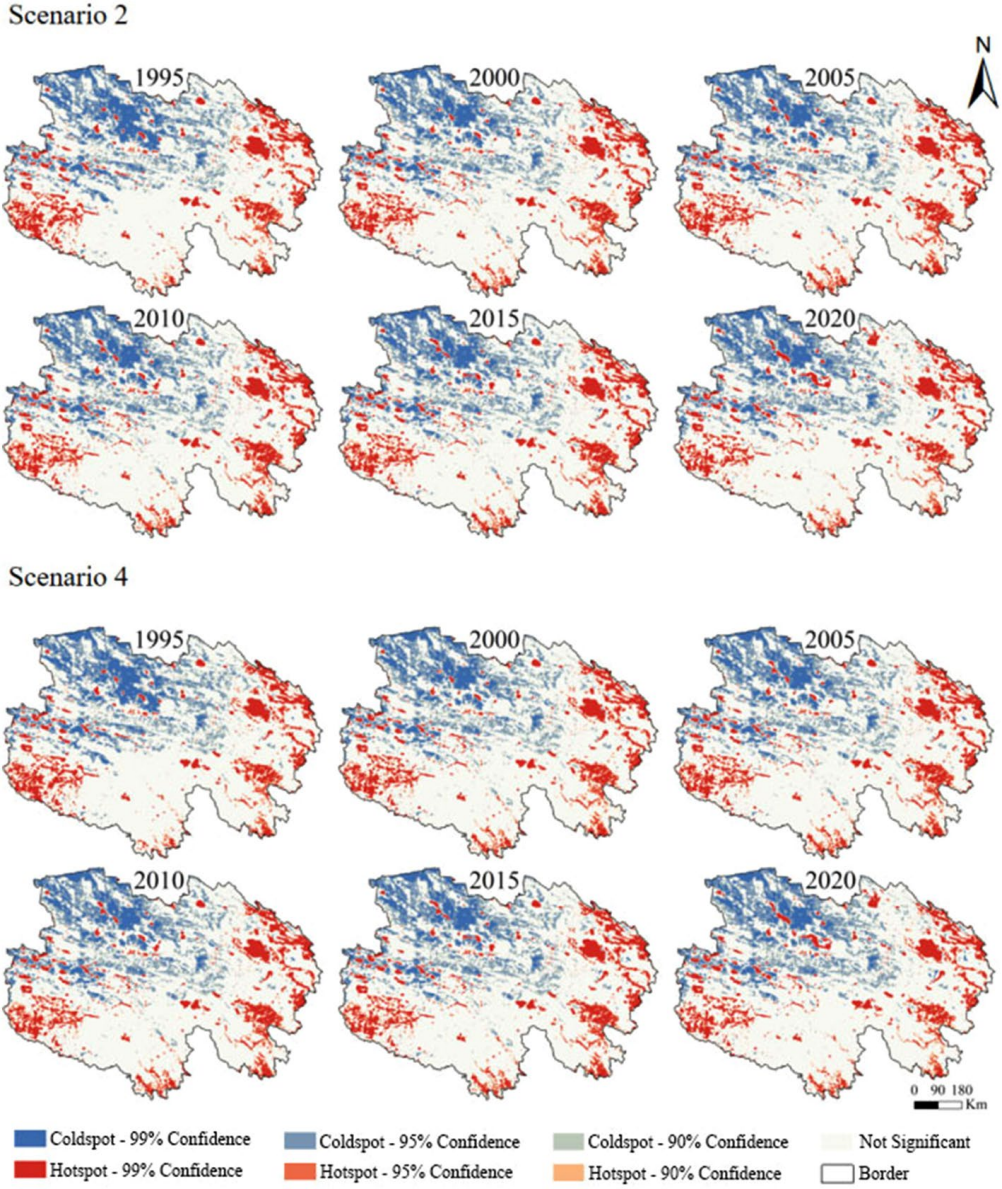
Spatial distribution of the coldspots and hotspots of ESSVs for scenario 2 and scenario 4 (made in ArcGIS 10.4.1, https://www.esri.com/zh-cn/arcgis/products/arcgis-desktop/resources).

The proportion of coldspots and hotspots can reveal changes in the spatial aggregation distribution of the ESSV. The spatial distribution of coldspots and hotspots of the ESSV under scenarios 2 and 4 from 1995 to 2020 are shown in Fig. 7. Overall, there was little change in the ratio of the areas of coldspots and hotspots of the ESSV between scenarios 2 and 4, and the dynamic changes were similar. In both scenarios, the total coldspots area increased, decreased, and increased, reaching the maximum value of 21.83% in 2010. In both scenarios, the total hotspots area increased before 2015, then decreased after 2015, and reached the maximum value of 14.54% in 2015.

The spatial distributions of the ESSV coldspots and hotspots were basically the same under two scenarios. The hotspots were principally concentrated in the southwest and east of Qinghai, where forest and water bodies were the major LULC types. The coldspots were principally concentrated in the northwest of Qinghai, where unused land was the dominant land use type.

## Discussion

There have been obvious changes in LULC since the GGP was launched in Qinghai. The forest area increased and the grassland area decreased in the years before GGP implementation. The forest area remained basically unchanged from 1995 to 2015 until it decreased from 2015 to 2020; the grassland area decreased slightly from 1995 to 2015 and increased significantly from 2015 to 2020. From 1995 to 2020, farmland converted into forest and grassland covered an area of 3,016 km^2^, and other land use types converted into forest and grassland covered an area of 83,204 km^2^. The area of unused land converted to grassland was the largest, reaching 75,617 km^2^. In terms of spatial distribution, forest and grassland areas were located all over the study area and were principally concentrated in the eastern and central parts of Qinghai. PD, LSI and SHDI values obtained from the analysis of landscape patterns showed similar changes. From 1995 to 2000 before GGP was implemented, PD, LSI, and SHDI values increased significantly, and there was strong human disturbance^71^. Since the GGP was implemented in 2000, in addition to 2015–2020, PD, LSI, and SHDI values increased slightly, indicating that the severity of landscape fragmentation in Qinghai gradually increased, and the degree of heterogeneity^72,73^, landscape patch irregularity, spatial heterogeneity, shape diversity, and land use richness also increased. The reason may be that there is a strong subjective arbitrariness in the spontaneous activities of the masses during the implementation of the GGP. The LPI value and the other three landscape index values showed opposite trends. The concentration of landscape types in Qinghai increased, which is associated with the land use type of the largest patch. From 1995 to 2020, the total annual NDVI of Qinghai continuously increased, which means that after implementation of the GGP, the vegetation improved. The western part of Qinghai was principally a vegetation degradation area, while the eastern part of Qinghai was principally a vegetation improvement area, and the Qinghai Lake area greatly improved. Overall, these results indicate that the increase of vegetation coverage in Qinghai was due to the GGP.

ES directly benefit human beings and are essential to economic development^74^. The ESV is affected by the fluctuation of supply and demand, and this study showed that implementation of the GGP can increase the effect of supply on the ESSV, and thus affect the total ESSV. According to scenario 1, changes in LULC changes affect the process and pattern of the ecosystem, and then change ES. These results are consistent with the conclusions of Akber et al.^75^. LULC changes can have an impact on ES. Different types of LULC can lead to differences in ES supply^76^; a change in land use significantly affects ES^77^, and the efficiency of ES is associated with land use intensity^78^. Implementation of the GGP can effectively increase the regional ESV. Analysis of land-use transfer types showed that LULC changes led to significant changes in the ESV. Since 2000, the ESV continued to increase, with a growth rate of 7.76%. The reason for this increase is an increase of grassland and water body area and a decrease of unused land and built-up land area. From 1995 to 2020, Qinghai's population increased by 32.37%, and the per capita GDP increased by about 42 times. The ESSVs in scenario 2 and scenario 4 continuously increased. The supply-driven change of the ESSV can be quantified by comparing scenarios 2 and 4. Before the GGP implementation, the ESSV of scenario 2 was greater than that of scenario 4; the growth rates of the ESSVs in scenario 2 and scenario 4 were 22.81% and 23.12% respectively; the effect of supply on ESSV is smaller than the effect of demand on ESSV. However, owing to the implementation of the GGP, the ESSV of scenario 4 became lower than that of scenario 2. From 2005 to 2010, the growth rate of the ESSV for scenario 4 was lower than that for scenario 2, and the increase in supply significantly inhibited the growth of the ESSV. After 2010, the growth rate of the ESSV for scenario 2 was slightly higher than that of scenario 4; the increased supply could not restrain the growth in demand; and the supply and demand of ES were extremely unbalanced. The economy is the leading factor affecting the ESSV. During the study period, the economy continued to increase, and the human demand for public services driven by economic factors inevitably increased simultaneously, but ES were not sufficient to meet the demand. Although the NDVI has increased and green space resources have been restored in Qinghai, which has alleviated the increase of the ESSV to a certain extent, it still cannot offset the increase in the ESSV caused by the increase in demand. Assessing the ESSV can provide information for making effective decisions regarding land management. The distribution of LULC types affects the distribution of coldspots and hotspots. The LULC types of hotspots were mainly forest and water bodies, and the LULC type of coldspots was mainly unused land in Qinghai. Along with the continuous improvement of the social economy in Qinghai, the demand for ES provided by forest and water bodies is increasing. Thanks to the GGP, forest and grassland areas in Qinghai are increasing, and the supply provided by forest and grassland is also increasing to alleviate people's demand for supply. Above all, the GGP can restrict the growth of the ESSV to a great extent.

Qinghai is an important area for the Belt and Road Initiative, and the Three-River Source and Qilian Mountain in Qinghai are important sources of fresh water resources in China^24^. Qinghai is the core area of Qinghai-Tibet Plateau and an important ecological security barrier in China^60^. In view of its unique geographical location and environmental conditions, Qinghai has an irreplaceable ecological position for continuously improving the quality of regional ecological environment and promoting the high-quality development of China. The stability of Qinghai's ecological environment is of great significance to the whole country and even Southeast Asia^24^, so it is not only the hub of China's ecological balance, but also the key area of GGP implementation. The innovation of this study lies in quantifying the impact of GGP on ESV in Qinghai. In addition, this study discusses the impact of GGP implementation on ESSV in combination with economic concepts. Therefore, this study fills the blank of this research direction in Qinghai. Studying the impact of GGP on ES in Qinghai can provide a basis for land management policies in the high-altitude river source areas, including Qinghai-Tibet Plateau. There are innovations in this research, but there are also some uncertainties and limitations. Taking into account the scope of Qinghai and the amount of work, when analyzing the land use dynamics, this paper selected the change in NDVI and Transfer Matrix Map values to represent the changes in LULC. During the research period, there was no obvious abrupt change in the climate of Qinghai, and the mechanism of vegetation's influence on the climate was complex. Vegetation growth could affect the regional climate and even the global climate through the exchange of material, energy and various biological processes between land and air^79,80^. In this paper, the contrastive analysis is selected to quantitatively analyze the changes of ESSV before and after GGP implementation. The results show that the supply level of ES has been significantly improved after the implementation of GGP, effectively alleviating the scarcity of ES in Qinghai. Besides GGP, Other ecological policies are implemented in several counties of Qinghai, but these ecological protection policies are carried out locally and sporadically. Although the factors that affect the experimental results are very complicated. However, there are many methods to evaluate the policy effect, and different methods also have their advantages and disadvantages. Undoubtedly, if more indicators representing natural and man-made factors are added to the study area, and various policy evaluation methods are used to quantify the changing trend of the research object, more accurate and convincing conclusions can be obtained, which also points out the direction for future related research.

It is shown that GGP can increase ESV and slow down the growth rate of ESSV. Therefore, the high-altitude river source areas, including the Qinghai-Tibet Plateau, should adhere to GGP. In view of the harsh natural conditions in some high-altitude areas and the high cost of project implementation, before the implementation of GGP, we should make a good plan according to local conditions, strengthen capital investment, and carry out regular maintenance after the completion of the project to maximize the benefits of GGP.

## Conclusion

As a large-scale ecological restoration project, the GGP has made outstanding contributions to the restoration of the harsh ecological environment in western China. Given the unique ecological environment and geographical location of Qinghai, it is important to clarify the influence of GGP implementation on the ESV and ESSV. This paper quantified and evaluated the land use dynamics, ESV, and ESSV in Qinghai from 1995 to 2020. The conclusions are as follows:

From 1995 to 2020, the total NDVI of Qinghai continued to rise, and the area converted to forest and grassland was the largest. The GPP changed the landscape pattern of Qinghai: landscape patch irregularity and spatial heterogeneity increased; the shape diversity, land use richness, and the concentration of landscape types increased; and the stability of landscape structure was enhanced. The vegetation status in Qinghai gradually improved. The coverage of green space in Qinghai Province increased gradually from northwest to southeast. In addition, the ESV decreased slightly from 1995 to 2000 and then increased significantly from 2000 to 2020 with LULC changes. From 1995 to 2020, the ESSVs of scenarios 2 and 4 increased. However, comparing the ESSVs of scenario 2 and scenario 4 revealed that the GGP increased the supply and alleviated the increase of the ESSV. The evaluation of the effect of implementation of the GGP in Qinghai can provide a theoretical basis for future policy and provide a concrete reference for the assessment of the ESV and ESSV.

## Data Availability

The datasets generated during and/or analysed during the current study are available from the corresponding author on reasonable request.
